# Influence of production process design on inclusion bodies protein: the case of an Antarctic flavohemoglobin

**DOI:** 10.1186/1475-2859-9-19

**Published:** 2010-03-24

**Authors:** Ermenegilda Parrilli, Maria Giuliani, Gennaro Marino, Maria Luisa Tutino

**Affiliations:** 1 Department of Organic Chemistry and Biochemistry, Università degli studi di Napoli Federico II - Complesso Universitario M.S. Angelo via Cinthia 4, 80126, Naples, Italy; 2 Facoltà di Scienze Biotecnologiche, Università degli studi di Napoli Federico II, Naples, Italy

## Abstract

**Background:**

Protein over-production in *Escherichia coli *often results in formation of inclusion bodies (IBs). Some recent reports have shown that the aggregation into IBs does not necessarily mean that the target protein is inactivated and that IBs may contain a high proportion of correctly folded protein. This proportion is variable depending on the protein itself, the genetic background of the producing cells and the expression temperature. In this paper we have evaluated the influence of other production process parameters on the quality of an inclusion bodies protein.

**Results:**

The present paper describes the recombinant production in *Escherichia coli *of the flavohemoglobin from the Antarctic bacterium *Pseudoalteromonas haloplanktis *TAC125. Flavohemoglobins are multidomain proteins requiring FAD and heme cofactors. The production was carried out in several different experimental setups differing in bioreactor geometry, oxygen supply and the presence of a nitrosating compound. In all production processes, the recombinant protein accumulates in IBs, from which it was solubilized in non-denaturing conditions. Comparing structural properties of the solubilized flavohemoglobins, i.e. deriving from the different process designs, our data demonstrated that the protein preparations differ significantly in the presence of cofactors (heme and FAD) and as far as their secondary and tertiary structure content is concerned.

**Conclusions:**

Data reported in this paper demonstrate that other production process parameters, besides growth temperature, can influence the structure of a recombinant product that accumulates in IBs. To the best of our knowledge, this is the first reported example in which the structural properties of a protein solubilized from inclusion bodies have been correlated to the production process design.

## Background

Protein over-production in *Escherichia coli *(*E. coli*) often results in formation of inclusion bodies (IBs). Aggregation most probably occurs as a consequence of interactions among the newly-formed folding intermediates which expose hydrophobic residues at their surface [1]. For a long time it was believed that IBs were compact, insoluble aggregates of misfolded proteins [2], remaining in the cell as biologically inactive deposits.

However, some recent reports have shown that the aggregation into IBs does not necessarily mean that the target protein is inactivated [3,4]. Structural data collected from many model proteins revealed the presence of significant proportions of native-like secondary structure in IBs proteins [5,6]. Consequently, it is not surprising that the analysis of the biological properties of IBs formed by enzymes demonstrated in some cases the occurrence of enzymatic activity inside the IBs [3,7]. These evidences introduced the concept that IBs are composed, at least partially, by functional polypeptides, whose deposition is necessarily driven by discrete aggregation determinants, that act irrespective of the global folding state of the protein [8]. It has been observed that IBs containing a high proportion of correctly folded protein can be easily solubilized under non-denaturing conditions [9] by using mild detergents or polar solvents, widely preserving the target protein folding.

The prevalence and extent of native structure and biological activity of IB proteins is variable depending on the protein itself, the genetic background of the producing cells and the expression temperature [6,10].

Flavohemoglobins (flavoHbs) have been identified in a number of bacteria and yeasts [11]. These proteins are characterized by a modular structure, where a N-terminal hemoglobin domain, displaying a classical three-over-three α-helical sandwich motif around a single heme b [12], is linked to a C-terminal FAD-containing reductase domain which resembles ferredoxin reductase [13]. The flavoHbs C-terminal domain binds NAD(P)H and transfers electrons to the heme in the globin domain via FAD [14,15].

It is generally believed that flavohemoglobins provide protection against NO and related reactive nitrogen species although the exact mechanism of action is still under debate [16-21].

A flavoHb encoding gene (*PSHAa2880*) was identified by *in silico *genome analysis of the Antarctic Gram-negative marine eubacterium *Pseudoalteromonas haloplanktis *TAC125 (*P. haloplanktis *TAC125) [22]. In the present paper, the recombinant production of the psychrophilic flavoHb (hereinafter called *Ph*flavoHb) in *E. coli *cells was carried out in several different experimental setups in order to identify the best production condition. Indeed, previously reported results on *E. coli *flavoHb demonstrated that the heterologous over-production of flavoHb may lead to host cell damage due to the action of flavoHb as a potent generator of products of oxygen radical partial reduction (i.e., superoxide and peroxide) [23-25]. Due to the expected toxicity of the recombinant product, flavoHb recombinant productions were carried out exploring several expression systems and/or microbial cell factories, with different results [26]. Amongst many other examples reported in literature, recombinant production of *E. coli *flavoHb in *E. coli *was obtained in absence of oxygen and in presence of nitrosating compound, an experimental setup in which the *hmp *gene expression is physiologic and the flavoHb activity is required [27].

Starting from the above information, in the present work, the recombinant production of the flavoHb from the Antarctic Gram-negative bacterium *P. haloplanktis *TAC125 [22] was performed in *E. coli *cells exploring some conditions differing in presence of a nitrosating compound and in O_2 _supply.

All production processes resulted in the accumulation of the recombinant protein in IBs, from which it was solubilized in non-denaturing conditions. Comparing structural properties of the solubilized *Ph*flavoHbs, i.e. deriving from the different production processes, our data demonstrated that the protein preparations differ significantly in the presence of cofactors (heme and FAD) and in their secondary and tertiary structure, demonstrating the impact of the specific production process design on the quality of inclusion bodies protein.

## Results

### Recombinant production of *P. haloplanktis *TAC125 flavohemoglobin in *E. coli *cells resulted in full deposition of the protein in the inclusion bodies

The *PSHAa2880 *gene was PCR amplified to suitably introduce *NdeI *and *SalI *restriction sites, and cloned into pET22b vector corresponding sites, thus generating the recombinant pET22b-*2880 *plasmid.

*E. coli *BL21(DE3) cells were transformed with the recombinant vector and, keeping in mind that proteins coming from psychrophilic micro-organisms are often characterized by a moderate to extreme thermal-lability [28], the production of the *Ph*flavoHb was carried out at 20°C. However, two different production process setups were explored. First, *E. coli *BL21(DE3)(pET22b-*2880*) recombinant cells were grown in a 7.5 L automatic fermenter, in which the recombinant mesophilic cells were grown aerobically at 20°C till the culture density reached the value of 0.6 OD at 600 nm. Induction was then performed by IPTG, in the following conditions: i) addition of heme and FAD precursors (i.e. D-aminolevulinic acid, FeCl_3_, and riboflavin); ii) addition of the nitrosating compound sodium nitroprusside (SNP), and iii) in microaerophilic conditions (dissolved oxygen tension always below 5% of saturation). Microareophilic conditions were achieved by stopping air supply during the next 16-18 hr of fermentation. The second experimental condition consisted in growing recombinant cells in shake flask at 20°C until the culture absorbance at 600 nm reached 0.6 OD, when the protein production was induced in the same conditions as in automatic fermenter but without SNP and in aerobic conditions.

Then, cells coming from the above production processes were analyzed looking for production and soluble/insoluble distribution of the recombinant protein by cell fractionation followed by SDS-PAGE analysis. Both production processes resulted in the total deposition of recombinant flavohemoglobin as cytosolic inclusion bodies (IBs), that were called flask-flavoHb IBs and ferm-flavoHb IBs if derived from cells grown in shake flasks or in fermenter, respectively.

### Recombinant *P. haloplanktis *TAC125 flavohemoglobin is solubilized from inclusion bodies by non-denaturing solutions

**Figure 1:**
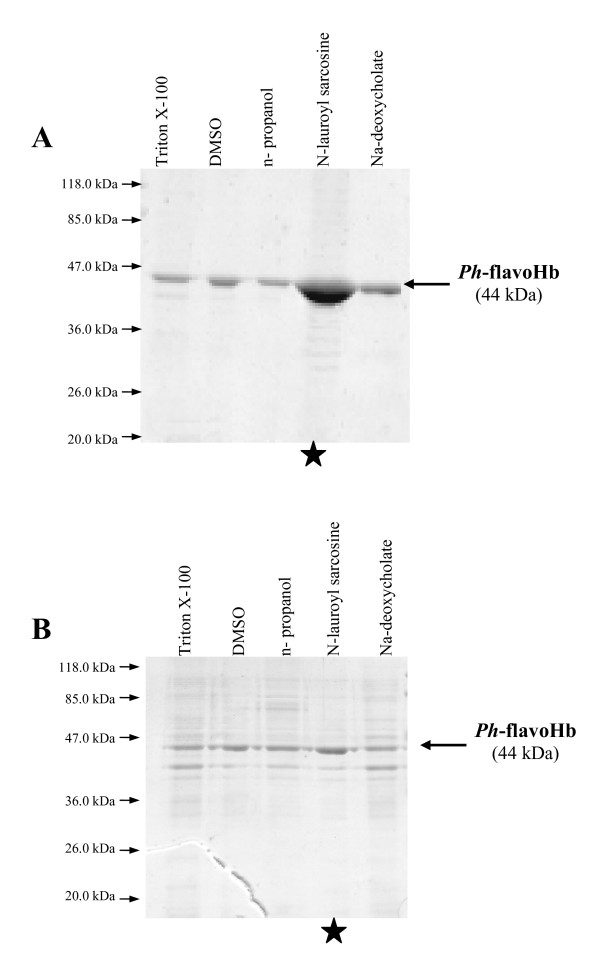
**Solubilization of flavoHb inclusion bodies produced in automatic fermenter (panel A) and shake flask (panel B)**. Same amounts of IBs were re-suspended in 40 mM Tris/HCl, pH 8.0 buffer containing different non denaturing agents. Same volumes of solubilized proteins were analysed by SDS-PAGE. Black star indicates that the loaded sample corresponds to one tenth of the other samples.

Treatment of *P. haloplanktis *TAC125 flavo-Hb IBs with different non-denaturing solvents such as low concentration of mild detergents or polar solvents was applied to the recovery of the recombinant protein in solution. Identical aliquots of flask-flavoHb IBs and ferm-flavoHb IBs were incubated overnight at 4°C with different non-denaturing solutions (i.e. buffered solutions containing 0.2% N-lauroyl sarcosine, or 5% DMSO, or 5% *n*-propanol, or 0.5% Triton X-100, or 1% Na-deoxycholate). Solubilized proteins were then separated from the insoluble matter by a centrifugation step and subjected to SDS-PAGE analysis. As shown in figure 1, both ferm-flavoHb IBs (panel A) and flask-flavoHb IBs (panel B) are partially solubilized in all tested conditions, although the solubilization yields (defined as the percentage of solubilized proteins relative to the total amount contained into the IBs sample) result to be quite different (Table 1). Indeed, best recovery in solution was obtained in N-lauroyl sarcosine either for ferm-flavoHb IBs or flask-flavoHb IBs (Table 1), but the corresponding solubilization yields exceed 95% in case of ferm-flavoHb IBs, while only about fifty percent of total proteins contained into IBs from flask culture went in solution. It is worth mentioning that in figure 1 the loaded amount of N-lauroyl sarcosine solubilized samples correspond to one tenth of the other samples.

**Table 1:** Percentage of solubilization of IBs in different solvents

	% solubilized protein	
**Solubilization solution**	**ferm-flavoHb**	**flask-flavoHb**

**0.5% Triton X-100 in 40 mM Tris/HCl, pH 8.0**	8 ± 0.6	10 ± 0.9

**5% DMSO in 40 mM Tris/HCl, pH 8.0**	9 ± 0.9	5 ± 0.4

**5% di n-propanol in 40 mM Tris/HCl, pH 8.0**	6 ± 0.7	5 ± 0.1

**0.2% N-lauroyl sarcosine in 40 mM Tris/HCl, pH 8.0**	95 ± 1.0	57 ± 0.8

**1% Na-deoxycholate in 40 mM Tris/HCl, pH 8.0**	22 ± 1.5	12 ± 0.3

Percentage of solubilization of IBs in different solvents was calculated using as 100% the protein concentration obtained dissolving IBs in urea 8 M and comparing this value with the protein concentration of samples obtained by treatment with different solvents. Evaluation of protein concentration was obtained by measuring the absorbance at 280 nm (Abs280).

The two solubilized IBs preparations also differ in their respective protein composition. As shown in figure 1, flask-flavoHb IBs seems to contain several proteins other than flavoHb (panel B), while IBs produced in fermenter contains almost only the psychrophilic recombinant protein (panel A).

### Structural comparison of recombinant *P. haloplanktis *TAC125 ferm- and flask-flavoHb

Flavohemoglobins extracted from either flask-flavoHb IBs (flask-flavoHb) or ferm-flavoHb IBs (ferm-flavoHb) were subjected to further analyses to investigate the presence of the two protein cofactors, i.e. heme and FAD.

**Figure 2:**
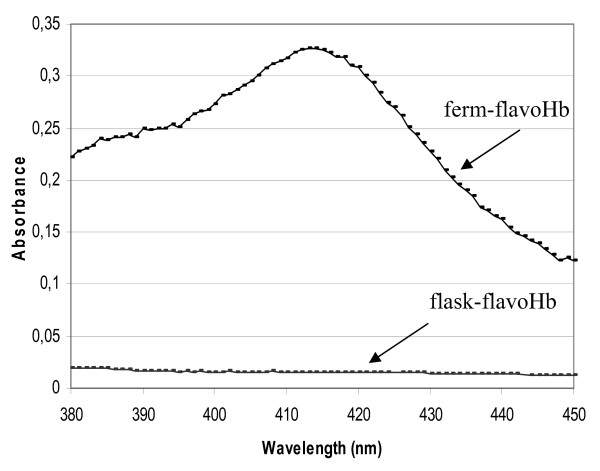
**Absorption spectra of ferm-flavoHb and flask-flavoHb**. Absorption spectra of cold-adapted flavohemoglobin extracted from IBs produced in fermenter (ferm-flavoHb) and in flask (flask-flavoHb). The spectra were recorded in 0.2% N-lauroyl sarcosine, 40 mM Tris/HCl pH 8.0, and the proteins concentration was 2 μM.

Absorption spectra of heme-containing proteins are characterized by the presence of Soret signal, a peak centred at about 413 nm. Therefore, UV/VIS absorption spectra of each solubilized flavoHb were recorded, and their respective spectra regions between 380 and 450 nm are shown in figure 2. Only the ferm-flavoHb spectrum is characterized by the presence of a typical Soret signal, centred at about 413 nm (Figure 2).

**Figure 3:**
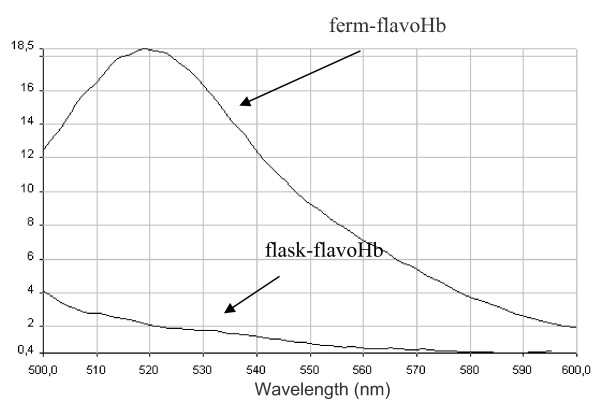
**Fluorescence spectra of ferm-flavoHb and flask-flavoHb**. Fluorescence spectra of cold-adapted flavohemoglobin extracted from IBs produced in fermenter (ferm-flavoHb) and in flask (flask-flavoHb). The spectra were obtained exciting at 450 nm and recording emission between 500 nm and 600 nm. The proteins were in 0.2% N-lauroyl sarcosine, 40 mM Tris/HCl pH 8.0, and their concentration was 2 μg/μl.

Then, the presence of the FAD cofactor in flask-flavoHb and ferm-flavoHb proteins was investigated by fluorescence measurements. In detail, emission spectra between 500 nm and 600 nm, exciting at 450 nm, were recorded and are shown in figure 3. An emission signal at 520 nm, which is indicative of the presence of the FAD cofactor, was only detected in the ferm-flavoHb fluorescence spectrum (Figure 3).

**Figure 4:**
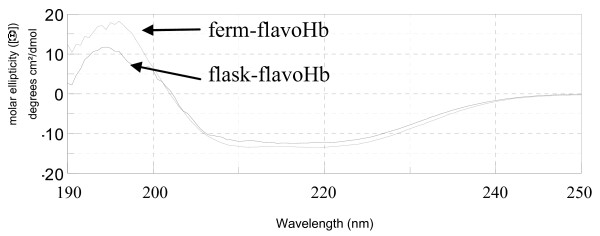
**CD analysis of ferm-flavoHb and flask-flavoHb**. CD analysis of ferm-flavoHb (A) and flask-flavoHb (B). The CD spectra were recorded in phosphate buffer 40 mM pH 8.0 at 25°C, the proteins concentration was 100 ng/μl.

To explore the secondary structure of flask-flavoHb and ferm-flavoHb proteins, circular dichroism measurements were performed. As shown in figure 4, both proteins display secondary structure, although not identical since the two recorded CD spectra are clearly not superimposable. The collected CD data were used to calculate the percentage of α-helix, β-sheets and random coil for each protein (by using the software K2d, accessible through the site http://www.embl-heidelberg.de/~andrade/k2d[29,30]). As shown in table 2, the two proteins differ significantly in their secondary structure content, and the ferm-flavoHb protein is predicted to have a higher content of either β-sheets or α-helix.

**Table 2:** Percentage of calculated secondary structures of ferm-flavoHb and flask-flavoHb

	Estimation of protein secondary structure from CD spectra by K2d	
	**ferm-flavoHb**	**flask-flavoHb**

**α-helix**	46%	39%

**β-sheets**	23%	17%

**random coil**	31%	44%

Percentage of secondary structures was calculated from CD data spectra by K2d software (Merelo, Andrade). K2d offers an algorithm for the estimation of the percentages of protein secondary structure from UV circular dichroism spectra using a Kohonen neural network

In case of ferm-flavoHb secondary structure prediction, the program has given a maximum error of 0.080. The maximum error obtained for flask-flavoHb secondary structure prediction is 0.085. This means that the sum of the errors in the prediction of the alpha, beta and random percentage values divided by three is expected to be less then 0.085. In both cases the error values are below the threshold maximal error 0.227. Maximal errors above this value indicate that the result given by the network prediction is not reliable.

**Figure 5:**
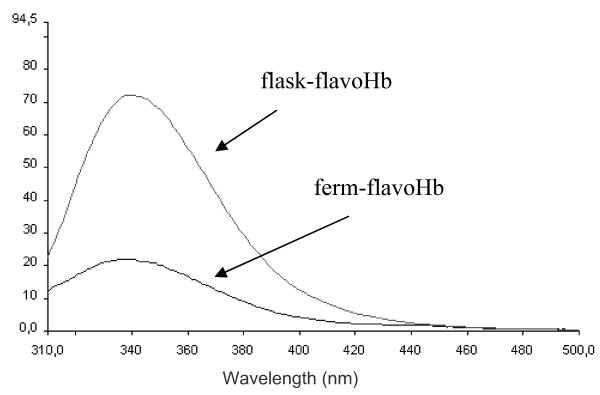
**Tryptophan fluorescence spectra of ferm-flavoHb and flask-flavoHb**. Fluorescence spectra of ferm flavoHb (A) and flask-flavoHb (B) obtained recording an emission spectra between 200 nm and 500 nm exciting at 295 nm. Fluorescence spectra were recorded in 0.2% N lauroyl sarcosine, 40 mM Tris/HCl pH 8.0, protein concentration was 2 μg/μl.

The structural comparison between flask-flavoHb and ferm-flavoHb proteins was then extended to the study of fluorescence emission spectra of tryptophan residues (3 Trp residues are present in the *Ph*flavoHb sequence). Emission spectra in the range between 310 nm and 500 nm, exciting at 295 nm, were recorded and are presented in figure 5. Both proteins display an emission spectrum λ_max _close to 338 nm, indicating that Trp residues are not solvent exposed. Both protein preparations were fully denatured by addition of guanidinium chloride (at a final concentration of 6 M) and the exposure of the tryptophan residues to a more polar environment was mirrored by the shift of the emission maximum to 366 nm (data not shown).

**Figure 6:**
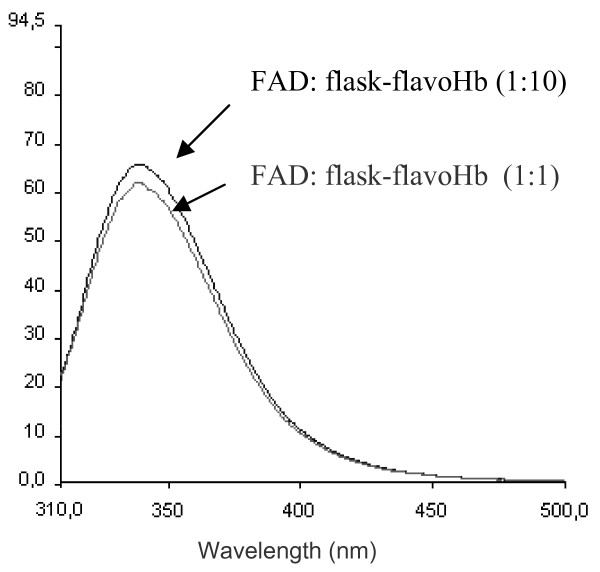
**Tryptophan fluorescence spectra of flask-flavoHb in presence of FAD**. Tryptophan fluorescence spectra of flask-flavoHb in presence of FAD cofactor. FAD was added to flask-flavoHb protein at molar ratio of 1:10 and 1:1. Fluorescence spectra were recorded in 0.2% N-lauroyl sarcosine, 40 mM Tris/HCl pH 8.0, proteins concentration was 2 μg/μl.

Spectra presented in figure 5 differ in the intensity of λ_max _signal, where flask-flavoHb protein has a fluorescence emission at 338 nm about three times higher than that of ferm-flavoHb. To investigate if the FAD cofactor, which is present only in ferm-flavoHb protein, is responsible for the observed quenching of the signal at 338 nm, tryptophan fluorescence spectra of flask-flavoHb were recorded in the presence of two molar ratio of exogenous FAD. As shown in figure 6, the intensity of λ_max _signal is not quenched by the addition FAD molecule, neither in the presence of the higher 1:1 FAD:protein molar ratio.

### Influence of different process parameters on inclusion bodies protein

**Figure 7:**
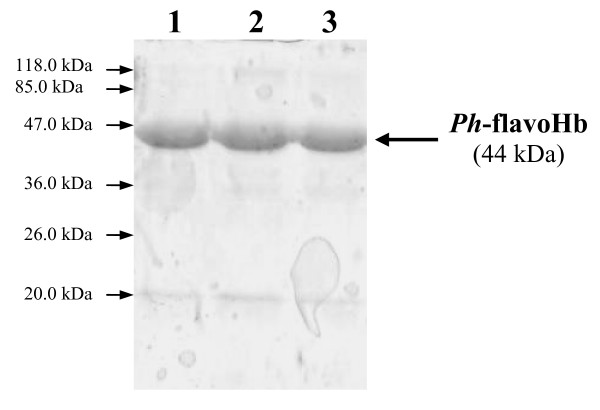
**FlavoHb inclusion bodies produced in automatic fermenter in different conditions**. SDS-PAGE of *Ph*flavoHb IBs extracted from culture obtained in microaerophilic conditions without SNP (1), in aerobic condition with (2) and without SNP (3). IBs were-suspended in 0.2% N-lauroyl sarcosine 40 mM Tris/HCl, pH 8.0.

In order to understand which process parameter is more relevant in flavohemoglobin production, i.e. if the observed differences between flask-flavoHb and ferm-flavoHb depend on the SNP presence, on the bioreactor geometry, or on oxygen availability, three different production process setups were explored. The production of the *Ph*flavoHb was carried out at 20°C in 7.5 L automatic fermenter, in microaerophilic conditions without SNP, in aerobic condition with and without SNP (always following the previously described induction conditions). Then, cells coming from the above production processes were analyzed and flavohemoglobin resulted to accumulate in inclusion bodies in all tested condition. *P. haloplanktis *TAC125 flavoHb IBs extracted from the different production processes were solubilized in presence of 0.2% N-lauroyl sarcosine. As shown in Figure 7, the solubilized IBs produced in fermenter contain almost only the psychrophilic recombinant protein indicating that the different protein composition of solubilized IBs of flask-flavoHb and ferm-flavoHb (Figure 1) was due to the bioreactor geometry, i.e flask or automatic fermenter. The analyses aimed to investigate the presence of the heme and FAD (data not shown) demonstrated that proteins obtained in microaerophilic conditions without SNP and in aerobic condition with and without SNP lack of both cofactors.

## Discussion

Several recent reports have shown that IBs could contain proteins that posses a native-like secondary structure and an enzymatic activity [3,5-7]. Moreover, it has been reported that IBs containing a high proportion of correctly folded protein can be easily solubilized under non-denaturing conditions [9]

In this paper we report a case in which using different process designs, we always obtained the complete accumulation of the recombinant proteins in IBs, from which the products were easily solubilized under non-denaturing conditions. Although their common attitude to get solubilized, recombinant proteins deriving from the different production processes differ in presence of cofactors and in their secondary and tertiary structure. In detail, we produced the flavohemoglobin from the Antarctic Gram-negative bacterium *P. haloplanktis *TAC125 [22] in *E. coli *cells in fermenter in the presence of a nitrosating compound (the SNP) and in microaerobiosis. In parallel, *Ph*flavoHb was produced in *E. coli *recombinant cells in standard conditions, i.e. grown in shake flasks by an aerobic production scheme and in absence of SNP. Both processes, carried out at 20°C, resulted in the total accumulation of the recombinant protein in cytoplasmic inclusion bodies. The treatment of ferm-flavoHb IBs and flask-flavoHb IBs with a N-lauroyl sarcosine solution highlighted unexpected differences in i) the solubilization yield, and ii) the composition of the solubilized matter. This evidence prompted us to carry out a structural comparison of the two recombinant *Ph*flavoHbs, in order to assess if the production setup could influence the main structural features of IBs proteins. Data reported in the present paper demonstrate that the two proteins differ significantly, mainly in the presence of FAD and heme cofactors. Indeed, when subjected to suitable spectroscopic analyses, proofs of the presence of heme and FAD were collected only in the case of ferm-flavoHb. Furthermore, CD spectra demonstrate that both proteins possess a secondary structure, but the ferm-flavoHb content of alpha-helix and beta-sheets is higher than that observed in the protein produced in flask.

Taking advantage from the presence of three tryptophan residues along the *Ph*flavoHb protein sequence, fluorescence emission spectra of tryptophan residues were recorded. Both spectra are characterized by a λ_max _emission signal around 338 nm, a result indicative that the Trp residues are not exposed to the solvent. Indeed, in these experimental conditions, unfolded proteins usually present a shift of λ_max_towards 350 nm. As expected, when the proteins were chemically denatured by guanidinium chloride, a shift of the λ_max _emission signal was observed (new maximum at 366 nm, data not shown). These data are highly suggestive that both proteins display a 3D structure, although likely not identical. In fact, fluorescence intensity at λ_max _of flask-flavoHb is about three times higher than that of the protein produced in fermenter. This different spectroscopic behaviour is not justified by the likely quenching effect of FAD cofactor associated only to the ferm-flavoHb. Therefore, the observed difference in intensity of λ_max _signal could be due to some amino acids residues close to tryptophan residues that work as fluorescence quencher in ferm-flavoHb. These results are suggestive of a different chemical surrounding around the tryptophan residues in flask-flavoHb and ferm-flavoHb proteins, thus supporting the conclusion that the two proteins differ in 3D structure too.

Furthermore, a systematic approach was applied to understand which process parameter was crucial for obtaining a protein endowed with FAD and heme cofactors. It turned out that the synergic effect of microareophilic growth conditions and the presence of SNP was essential. This experimental setup likely mimics the physiologic conditions in which flavoHb activity is required. The occurrence of a suitable physical-chemical environment and/or the induction of specific protein chaperones could justify the unique cofactors incorporation observed.

Without a structural/functional characterization of native *Ph*flavoHb, a final assessment on the quality of the recombinant proteins produced in this work cannot be formulated. However, the presence of FAD and heme cofactors, together with the collected indirect evidences of a different secondary and, eventually, tertiary structures, looks very promising of a better quality of flavoHb obtained in the presence of a nitrosating compound and in microaerobiosis.

## Conclusions

The present paper describes the recombinant production of a flavohemoglobin, a multidomain protein requiring FAD and heme cofactors for its activity. Two significantly different production process designs were explored, both resulting in the full product accumulation in IBs. Data reported here demonstrate that other process parameters, besides growth temperature, influence the quality of a recombinant product even if it accumulates in IBs. To the best of our knowledge, this is the first reported example in which the quality of protein solubilized from inclusion bodies has been correlated to the production process design.

## Methods

### Bacterial strains, plasmid and culture condition

The *E. coli *BL21(DE3) (Novagen) strain was routinely used for cloning and expressing recombinant gene. Cells were grown in Luria-Bertani (LB) medium at 20°C. When required, ampicillin (Sigma) was added at 100 μg/ml. Plasmid pET-22b (Novagen) was utilized for cloning and expression. Restriction and modifying enzymes were obtained from Promega. The oligonucleotides were custom synthesized from PRIMM.

### Cloning of the PSHAa2880gene

The primer pairs for the *PSHAa2880 *gene (Oligo 2880 fw 5' TTCATATGTTATCTGATAAAACTATTGAAA 3', Oligo 2880 rv 5' AAGTCGACTTATAGATCTTGATGCGG 3') were designed on the basis of the *P. haloplanktis *TAC125-genome sequence [31]. Sequences corresponding to the *Nde*I site and a *Sal*I site were introduced in the forward and reverse primers, respectively. The amplifications were performed in a mixture containing 80 ng of *P. haloplanktis *TAC125-genomic DNA as template, 50 pmol of each oligonucleotide primer, 1.8 mM MgCl2, 50 mM KCl, 20 mM Tris-HCl pH 8.3, 0.1% gelatine, 200 μM dNTP in a final volume of 50 μl. The mixtures were incubated at 95°C for 10 min, then 1.25 units of *Taq *DNA polymerase were added. Twenty cycles of amplification (consisting of 1 min at 95°C, 1.5 min at 60°C and 1 min plus 5 sec/cycle at 72°C) were carried out and followed by a cycle in which the extension reaction at 72°C was prolonged for 15 min in order to complete DNA synthesis. The amplified fragment was cloned and its nucleotide sequence checked to rule out the occurrence of any mutation during synthesis. The *Nde*I-*Sal*I-digested fragment of the *PSHAa2880 *gene was then subcloned into the corresponding sites of the expression vector pET-22b, obtaining the plasmid pET22b-*2880*. The recombinant vector was used to transform *E. coli *BL21(DE3) cells, that were used for the following production processes. All DNA manipulation were performed as previously described [32]

### Shake Flask Culture

For the over-expression of cold-adapted flavoHb in flask, a single colony of recombinant *E. coli *BL21(DE3) (pET22b-*2880*) was inoculated in LB medium supplemented with 4 g/L glucose and ampicillin (100 μg/ml) and allowed to grow at 20°C, in the rotary shaker, until absorbance at 600 nm reached ~0.6 OD. The culture was then induced with 1 mM isopropyl-β-D-thiogalactopyranoside and further incubated for another 16-18 h at 20°C in the presence of 50 μM of D-aminolevulinic acid, 3 μM FeCl_3_, 100 μM riboflavin. After production, the cell culture was aliquoted, centrifuged, and the supernatant was discarded. The bacterial pellet was stored for further analysis.

### Laboratory Fermentation

A proper preinoculum of overnight grown recombinant *E. coli *BL21(DE3) (pET22b-*2880*) was diluted in 4.5 L of LB medium supplemented with 4 g/L glucose and ampicillin (100 μg/ml) in a 7.5-L Techfors *S *(Infors, HT Switzerland) automatic fermenter. Cells were grown aerobically at 20°C till the culture density reached the value of 0.6 OD at 600 nm. Induction was then performed by 1 mM IPTG, in the presence of 50 μM mM D-aminolevulinic acid, 3 μM FeCl_3_, 100 μM riboflavin and 0.4 mM sodium nitroprusside (SNP). Then, air supply was stopped during the next 16-18 hr of fermentation keeping a microaerophilic conditions (dissolved oxygen tension always below 5% of saturation).

In case of production of the *Ph*flavoHb in aerobic condition with and without SNP the preinoculum of overnight grown recombinant *E. coli *BL21(DE3) (pET22b-*2880*) was diluted in 2.5 L of medium and dissolved oxygen tension was maintained always above 30% of saturation.

After production, the cell culture was collected, centrifuged, and the supernatant was discarded. The bacterial pellet was stored for further analysis.

### Protein inclusion bodies extraction

Biomass was harvested at the end of the production process by centrifugation, and the wet bacterial pellet was re-suspended in 10 mM Tris/HCl, pH 8.0. Samples were kept on ice and disrupted by sonication using an Branson sonicator (Model B-15), using a program consisting of 20 cycles (30" on, 60" off, intensity 4.5). After disruption of the cells, samples were centrifuged at 5000 rpm for 30 min at a constant temperature of 4°C. The supernatant was discarded, and the inclusion bodies fraction was washed twice with chilled water, divided in several aliquots and incubated overnight for the solubilization at 4°C in 40 mM Tris/HCl, pH 8.0 buffer containing alternatively:

• 5% n-propanol;

• 0.5% Triton X-100;

• 5% DMSO;

• 1% Na-deoxycholate;

• 0.2% N-lauroyl sarcosine.

As negative control the insoluble matter was treated with water. After the incubation, the suspensions were centrifuged at 4400 × ***g ***for 15 minutes at 4°C. The supernatants were analyzed by SDS-PAGE.

### Protein concentration measurements

The Bradford method [33] was applied to determine protein concentration. In case of protein solubilized from IBs with different solvents a qualitative measure of protein content was determined by measuring the amount of light absorbed at 280 nm (Abs280).

### Spectroscopic Measurements

UV-Vis absorption spectra were recorded in UNIKON 930 spectrophotometer. Fluorescence measurements were carried out in a PERKIN ELMER LS 50B fluorospectrometer. Circular dichroism (CD) spectra were obtained in a Jasco spectropolarimeter (model J-715) equipped with a thermostatically controlled cell holder.

## Competing interests

The authors declare that they have no competing interests.

## Authors' contributions

EP and MG performed the experiments and helped to draft the manuscript. EP and MLT drafted the manuscript and designed and coordinated the study. GM has been involved in manuscript preparation and critical reading. All authors read and approved the manuscript.
